# A Perspective Review on the Role of Nanomedicine in the Modulation of TNF-TNFR2 Axis in Breast Cancer Immunotherapy

**DOI:** 10.1155/2019/6313242

**Published:** 2019-05-23

**Authors:** Mohammad A. I. Al-Hatamleh, Suhana Ahmad, Jennifer C. Boer, JitKang Lim, Xin Chen, Magdalena Plebanski, Rohimah Mohamud

**Affiliations:** ^1^Department of Immunology, School of Medical Sciences, Universiti Sains Malaysia, Kelantan 16150, Malaysia; ^2^Department of Immunology and Pathology, Monash University, Melbourne 3168, Australia; ^3^School of Chemical Engineering, Universiti Sains Malaysia, Penang 14300, Malaysia; ^4^State Key Laboratory of Quality Research in Chinese Medicine, Institute of Chinese Medical Sciences, University of Macau, Macau SAR 999078, China; ^5^School of Health and Biomedical Sciences, RMIT University, Bundoora 3083, Australia; ^6^Hospital Universiti Sains Malaysia, Universiti Sains Malaysia, Kelantan 16150, Malaysia

## Abstract

In the past decade, nanomedicine research has provided us with highly useful agents (nanoparticles) delivering therapeutic drugs to target cancer cells. The present review highlights nanomedicine applications for breast cancer immunotherapy. Recent studies have suggested that tumour necrosis factor (TNF) and its receptor 2 (TNFR2) expressed on breast cancer cells have important functional consequences. This cytokine/receptor interaction is also critical for promoting highly immune-suppressive phenotypes by regulatory T cells (Tregs). This review generally provides a background for nanoparticles as potential drug delivery agents for immunomodulators and further discusses in depth the potential of TNF antagonists delivery to modulate TNF-TNFR2 interactions and inhibit breast cancer progression.

## 1. Introduction

The term “nanotechnology” is a concept that has only emerged in the last decade with the prefix “nano” cited from the Greek word “nanos”, indicating that something is dwarf-sized. Therefore, the term “nanotechnology” refers to a technology that uses very small particles invisible to the naked eye [1]. Before the 19^th^ century, although the term nanotechnology had not yet been globally defined, the applications of nanotechnology were already used in the industrial field, [2]. During a meeting of American Physical Society in 1959, for the first time, Richard Feynman discussed the term of nanotechnology systematically, laying the foundations of the nanotechnology field [3]. Subsequently, at the end of 19^th^ century and early of the 20^th^ century, the field of nanotechnology experienced a massive expansion, when almost all industrialised countries started pursuing nanotechnology research in all fields including medicine [4]. Introduction of modern nanotechnology in the medical field aimed at better prevention, diagnostics, and therapy of diseases and was later called “nanomedicine”.

**Figure 1:**
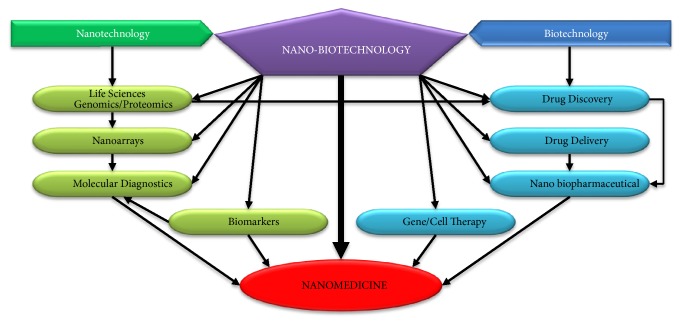
Illustration of how nanomedicine research is based on the applications of nanobiotechnology (adapted from Jain, 2008 [5]).

Nanomedicine is a new science that emerged along with the establishment of technologies such as high resolution microscopes for biotechnology applications that allow investigations of nanomaterials (less than 100 nm) at cellular levels (Figure 1) [5]. Among several different nanomedicine platforms, nanotechnology-based drug delivery has received the greatest interest. Incorporating therapeutic drugs into nanomaterials and using these as carriers to target specific tissues, avoiding systemic side effects, remains a major challenge in therapeutics [6, 7]. Many types of nanocarrier systems from diverse materials with distinctive physiochemical properties have been established for use in multiple diseases (Table 1), including the most common and explored type, liposomal drug carrier systems [8].

As cancer is one of the biggest health challenges facing humanity, a substantial amount of research has focused on nanomaterials as drug delivery agents to target cancer tissues, as illustrated by almost 12,000 manuscripts in the recent decade [33]. However, interest among the researchers in applying nanomedicine applications in different cancer types has varied with breast cancer receiving the least attention from nanomedicine, despite the fact that it is the most globally widespread cancer type with alarming rates of occurrence in many countries [34]. Furthermore, the majority of these studies used nanomaterials to target cancer cells with chemotherapy/drugs, while few studies focused on the use of nanomaterials to treat/control breast cancer in the context of immunotherapy. The most recent study used gold nanoparticles in breast cancer cells to deliver* Commiphora myrrha* and* Boswellia sacra* extracts to induce trisodium citrate dihydrate reduction which leads to cytotoxicity in breast cancer and normal cells. The study reported cytotoxicity in breast cancer cells, but no harm in normal breast cells [35].

Tumour necrosis factor (TNF) is generally considered a master proinflammatory cytokine [34]. During inflammatory processes (including the cancer microenvironment) TNF is one inflammatory mediator that is produced secreted firstly [37]. It fosters the generation of a cytokine cascade and promotes the production of other inflammatory mediators [e.g., transcription factors, interleukin (IL)-1, IL-6] [38, 39]. There are two types of TNF receptors (TNFR1 and TNFR2) localised at the cellular surface, which have unrelated intracellular regions [40]. A study in a model of inflammation-associated cancer revealed that TNFR2 is preferentially upregulated over TNFR1 and that treatment with the anti-TNF monoclonal antibody reduced the number and size of tumours [41]. Therefore, TNF-TNFR2 axis was implicated in the suppression of immune response and affects tumour progression and metastasis [42]. In the following sections, we will interpret a possible application of targeting TNF-TNFR2 interactions using a nanomedicine platform in breast cancer. This neutralisation of TNF as well as TNFR2 by using TNF antagonist drugs delivered through nanoparticles might be an effective therapeutic strategy on breast cancer cells. To the best of our knowledge, this is the first article discussing this hypothesis.

## 2. Nanomedicine and Breast Cancer

**Figure 2:**
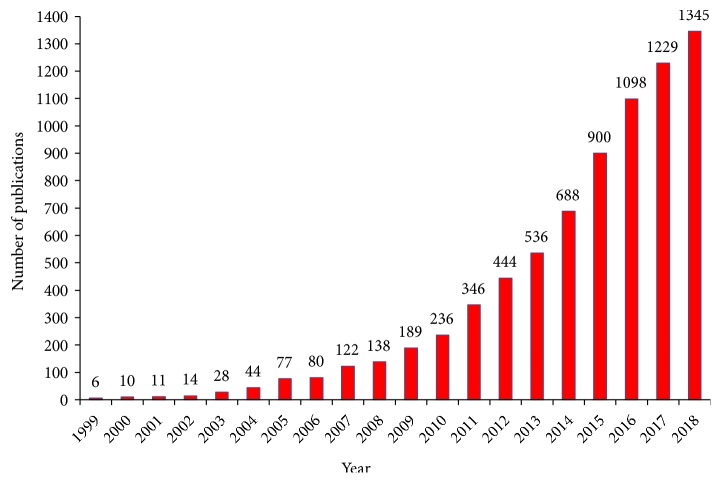
Annual publications regarding cancer nanomedicine research in the recent 20 years (applied on PubMed database on December 11, 2018, by using search terms: cancer nanomedicine/nanoparticles).

Cancer includes a range of diseases with alterations in the biological status of any nucleated cells, which causes malignant tumours with abnormal growth and division (neoplasia) [43]. It is one of the biggest challenges facing the world and is causing huge continuous losses without reaching effective-comprehensive solutions [43, 44]. Currently, both medical and research community have attempted an approach to nonconventional cancer therapies that can limit damage or loss of healthy tissues and be able to fully eradicate the cancer cells. Nanomedicine represents an efficient drug delivery system, which can deliver therapeutic agents directly to the targeted cancer cells only and minimize the dose-dependent side effects of drugs on nontarget sites [45]. By focusing on the targeted site, this could result in enhanced drug efficiency compared to conventional chemo/radiotherapy [46, 47]. Furthermore, the growing interest in utilizing this application for cancer research has been significantly increased year by year (Figure 2).

**Figure 3:**
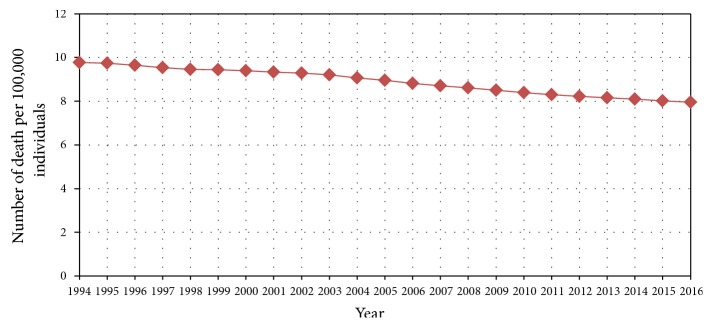
Global death rates caused by breast cancer between 1994 and 2016 [36].

**Figure 4:**
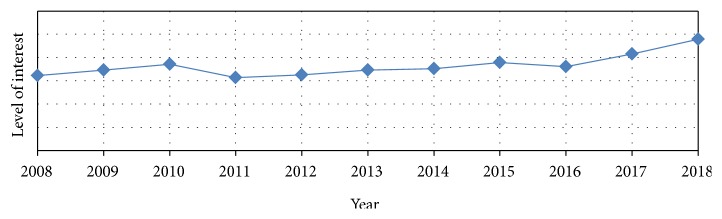
A search shows the levels of population awareness regarding breast cancer medications/treatments, over the recent 10 years. Applied on the Google Trends database up to December 11, 2018.

In 2016, the global prevalence of cancer ranged from 0.2 to 2 percent approximately [36]. Breast cancer was reported as the highest cancer prevalence with 0.12 percent, and until 2016 there were a total of 8.15 million breast cancer cases [36]. There was more than 20% increase in the global prevalence rate of breast cancer up to 10 years from 2008 to 2017 [48]. Moreover, breast cancer was classified in 2018 as the most common cancer among women, and the second most widespread cancer with more than 2 million cases diagnosed over the world [49]. According to the Avon Breast Cancer Foundation, in 2002, there were over 39,600 deaths caused by breast cancer among American women only [50]. Although breast cancer prevalence rates are increasing continuously, recent statistics have reported a decline in death rates (Figure 3) [36]. This decline could be due to increased awareness about the preventive measures and the periodic and early detection as well as treatment (Figure 4).

There are various complex classifications for breast cancer; the best one is the molecular phenotype classification that includes five different subtypes based on cancer genes expression such as molecular markers (Table 2) [51, 52]. The treatment and its effectiveness between various breast cancer types are different, and once metastasized, the effectiveness of all treatment strategies will be reduced [53]. Therefore, search for a more effective therapeutic option has been highly anticipated, especially in breast cancer.

In recent years, along with the extensive identification of molecular markers on breast cancer, several novel nanomedicine applications have been developed to specifically target these pathways (Table 3). Targeting breast cancer cells involves attaching specific molecules (ligands) on the surface of nanoparticles, and these ligands are able to recognize and bind only to complementary molecular markers found on the surface of targeted breast cancer cells [54]. Ligand-nanoparticle conjugate binds to the receptors (e.g., HER-2, EGFR, VEGFR, IGF-IR) expressed on the breast cancer, mediates internalization of nanoparticles through endocytosis, and releases the conjugated biomolecules by lysosomal degradation to the active sites of tumour cells [54]. As reviewed below, TNFR2, an immune checkpoint stimulator and oncogene, has more recently emerged as a potential new target for breast cancer therapeutics via its modulation on TNFR2 [55]. However, to date there is no study focusing on the development of nanomedicine targeting TNF-TNFR2 axis for breast cancer therapeutics. Generally, TNF-TNFR2 axis plays a significant role in the overall regulation of regulatory T cells (Tregs), providing protection for cancer cells by promoting their immune evasion in an immunosuppressive environment [55], besides activating myeloid-derived suppressor cells (MDSCs) to enhance tumour immune escape [56, 57].

## 3. TNF-TNFRs Interactions

TNF is a multifunctional cytokine secreted by various types of cells as well as being responsible for leukocyte recruitment, monocyte chemoattraction, and increased regulation of adhesion molecule expression and may also promote apoptosis [68]. TNF is expressed by immune cells including activated macrophages/monocytes, activated T cells, and natural killer (NK) cells and could be expressed by other nonimmune cells (e.g., fibroblasts and endothelial cells) [69]. The complexity of understanding the roles of TNF is partially due to the presence of different forms of TNF with equally different roles [70]. The membrane-bound form of TNF (mTNF) or pro-TNF is a transmembrane protein of 26 KDa which later can be converted to a soluble form of TNF (sTNF) which is released when mTNF is cleaved by TNF-converting enzyme (TACE) [71]. Although sTNF is the first to be in charge of the majority of responses, some studies have reported that mTNF has also the capacity to mediate similar responses, including some inflammatory responses, proliferation, B cells activation, and apoptosis [72]. Moreover, it was reported that the biological action of mTNF is based on cell contact-dependent signals. For example, mTNF has been shown to mediate inflammatory responses in astrocytes, but not in neurons, whereas both cell types sTNF have similar proinflammatory effects [73].

Both sTNF and mTNF are regulated by binding with their two receptors localised at the cellular surface. TNFR1 (p55) is encoded by* TNFR1* gene located on chromosome 12p13.31, consisting of 10 exons, and codes for a 55/60 kDa membrane receptor. TNFR2 (p75) is encoded by* TNFR2* gene located on chromosome 1p36.22, consisting of 10 exons, and codes for a 75/80 kDa membrane receptor [74]. These two receptors mediate different biological activities from TNF [75]. Studies have shown that the affinity of TNF for TNFR1 is lower compared to TNFR2; therefore, TNFR1 binds preferentially to high TNF concentrations and vice versa for TNFR2 [76, 77]. TNFR1 is expressed in nearly all nucleated cells, although in low levels [78]. Also, TNFR1 has been reported to be the primary mediator of TNF-induced apoptosis, linked to an intracellular region of TNFR1 called “death domain (DD)” that activates the nuclear factor kappa B (NF-*κ*B) pathway [79]. Activation of NF-*κ*B pathway plays a key role in the expression of genes that are responsible for encoding antiapoptotic proteins and several proinflammatory cytokines, including TNF, IL-6, and IL-1 [80]. On the other hand, studies showed that mTNF preferentially binds to and activates TNFR2, while sTNF binds to and activates TNFR1 [81]. Furthermore, TNFR2 participates in activation of B cells, enhances apoptosis by TNFR1, and plays a key role in other proinflammatory responses, including proliferation of T cells [80].

Upon binding of TNF to TNFR1, TNFR1 interacts with receptor-interacting protein 1/2 (RIP1/2) and TNFR1-associated DD protein (TRADD) to build a receptor complex [82] that induces Fas-associated DD protein (FADD), resulting in apoptosis [83]. However, TNFR1 is also able to induce other adaptor molecules which enhance cell survival, including cellular inhibitor of apoptosis protein 1/2 (cIAP1/2) and TNFR-associated factor 1/2 (TRAF1/2). These antiapoptotic signals by both cIAP1/2 and TRAF1/2 are acquired via downstream activation of NF-*κ*B pathway [84]. On the other hand, studies found that TNF and TRAF3 are necessary for activated T cells [85]. The expression of the full-length isoform of TRAF3 lacking exon 8 (Traf3DE8) allows the activation of noncanonical NF-*κ*B pathway by the deactivation of the NF*κ*B-inducing kinase (NIK)-TRAF3-TRAF2 axis, which results in aggregation of NIK in activated T cells [84]. Noncanonical NF*κ*B signalling pathway in turn regulates expression of some chemokines needed in adaptive immunity and structuration of the secondary lymphoid organ, such as B cell chemoattractant (CXCL13) [86]. Although the process(es) that drives the differential regulation of the alternatively spliced form of TRAF3 is not totally clear yet, some studies have reported that T cell-specific TRAF3^−/−^ mice were able to double the number of normal TNFR2-expressing Tregs [87]. Tregs, positive for CD4, CD25, and Foxp3, primarily suppress excessive inflammation [88], and expression of TNFR2 on Tregs identifies them as highly suppressive Tregs [89]. Therefore it could be highly beneficial to use TNFR2 as a potential target in cancer therapeutics [89–92].

The implication of TNF in almost all steps of tumourigenesis has been reported, both as an angiogenic and antiangiogenic factor, depending on the TNF doses and nature (soluble and membrane-bound) [93]. Since TNFR1 and TNFR2 differ in their cytoplasmic domain, they trigger distinct signalling pathways [i.e., proapoptotic (TNFR1) and prosurvival (TNFR2)] upon interaction with TNF [94]. In recent years, several studies on different types of tumours have reported a high expression of TNFR2, resulting in enhanced proliferation, angiogenesis, and migration of several tumour types [95]. This enhancement of tumourigenesis by TNFR2 is coordinated through the stimulation of NF-*κ*B or AKT serine/threonine kinase 1 signalling pathways, which in turn regulate DNA damage and repair of poly (ADP-ribose) polymerase (PARP) protein [96]. Moreover, preclinical studies found that blocking TNFR2 is sufficient to reduce the development of TNF-activated cells [97] as well as to increase TNF-associated cancer cell death [98]. TNFR1 shows high affinity to both soluble and membrane-bound TNF, while TNFR2 is only fully activated by mTNF [99]. Due to their different structure, their regulation through signalling pathways (MAPK and NF-kB) would induce different effects. TNFR1 is responsible for apoptosis while TNFR2 is responsible for cell proliferation and survival [100]. However, under some conditions, prolonged cell stress or disease condition, shift of TNFR2 to TNFR1 apoptotic signalling could occur [39]. All together these findings partially elucidate the role of TNFR2 in development of cancer and its differential function compared to signal kinase activation through TNFR1.

## 4. An Implication of TNF-TNFR2 in Breast Cancer

Numerous studies have explored the association of TNF and its receptors in breast cancer progression as well as the therapeutic possibilities. However, only a few investigated the impacts of TNFR2 expression in breast cancer [101]. In 2008, for the first time Rivas et al. studied the implication of TNF and its receptors (TNFR1 and TNFR2) on the molecular mechanisms and intracellular pathways of breast cancer proliferation [97]. This study showed that TNF enhances proliferation of breast cancer cells via the activation of p42/p44 mitogen-activated protein kinases (MAPK) pathway by binding to both TNFR2 and TNFR1. In addition c-Jun N-terminal kinase (JNK) and phosphoinositide 3-kinase (PI3K)/AKT pathway activation was also involved while NF-kB transcriptional activation was acquired by TNFR1 activation only [97]. However, in 2017, Yang and his colleagues showed that TNFR2 was implicated in promoting the progression of breast cancer via stimulation of AKT signalling pathway [95]. This signalling pathway protects cancer cells against DNA damage, which in return enhances breast cancer cell proliferation, cancer-associated fibroblast (CAF) induction, angiogenesis, and carcinogenesis [95]. In another study, Yang and his colleagues were able for the first time to confirm that there was a positive association between TNFR2 expression and its prognosis in terms of size of tumour, higher pathological grade, and advanced clinical stage [102]. They reported that the expression levels of TNFR2 in breast cancer cells were positively associated with doxorubicin (anthracycline type of chemotherapy) resistance; overexpression of TNFR2 significantly promoted doxorubicin resistance, while less expression of TNFR2 significantly dampened doxorubicin resistance, while in turn this regulated the DNA damage and repair PARP protein [95]. In 2018, Nie et al. used two types of antibodies: a TNFR2-blocking and a CD25-targeted approach as a combination treatment in a colon cancer mouse model and breast cancer mouse model, resulting in the inhibition of cancer progression in both models [103]. As per our knowledge, no study examined the expression of TNFR2 in breast normal cells, while we found only one study reporting that it was detected at low levels in normal vascular endothelial cells [104].

As TNFR2 exists without DD, it can enhance proliferation and activation of Tregs via 3 main pathways, namely, NF-kB, activator protein 1 (AP1), and MAPK pathways [105], therefore avoiding the immunosuppressive effect of TNF which is similar to cancer cells survival pathways [100]. Studies demonstrated that Tregs expressed higher levels of TNFR2 than any other T cells, and these high expression levels by Tregs were correlated with the most suppressive population [89]. Moreover, a study performed by van der Most et al. in 2009 [106] used cyclophosphamide to downregulate Tregs during chemotherapy for cancers, as Tregs depletion could be used to enhance the effectiveness of chemotherapies. They also reported that the drug gemcitabine depleted cycling Tregs concurrently with downregulation of CD4+ CD25+ T cells [106]. In addition, few studies also showed that TNFR2 also inhibits the antitumour role of effector T cells (Teffs) and decreases cancer immune responses [107]. Torrey et al. proved that targeting TNFR2 could be an effective treatment as TNFR2 antagonistic antibodies inhibit proliferation of both cancer cells and tumour-infiltrated Tregs while inducing the expansion of Teffs [108]. Furthermore, study in both colon and breast cancer models shows that combination of immunotherapeutic stimulants with TNFR2-blocking antibodies not only inhibits the proliferation of cancer cells but also decreases the number of Tregs and the surface abundance of TNFR2 on Tregs, thus enhancing the effectiveness of treatment [103]. However, to date in addition to their impact on both Tregs and Teffs activities in breast cancer microenvironment, no study has examined the effectiveness of nanomedicines targeting TNF receptors for ligand-nanoparticle conjugate or using TNF antagonists (e.g., biomolecules) as a potential therapy for breast cancer in humans.

It has previously been shown that TNF antagonism (anti-TNF) is a successful therapeutic option that has been applied in various inflammatory cases, including inflammatory bowel disease (IBD), spondyloarthritis (SpA), psoriasis, and rheumatoid arthritis (RA) [109]. TNF antagonism prevents ligand triggering of TNF-TNFRs signalling and thus blocks TNF's cytotoxicity and inflammatory capacity [110]. Currently, there are five approved TNF antagonists used to treat symptoms in inflammatory disorders, including Etanercept, Infliximab, Adalimumab, Certolizumab Pegol, and Golimumab [109]. Among them, Etanercept is a novel TNFR2:IgG1 fusion protein that was developed and approved by FDA in 1998 [111], and it is the only TNF antagonist that is a nonmonoclonal antibody and does not contain a fragment crystallisable (Fc) portion, which means that it is unable to encourage complement activation, antibody-dependent cell-mediated cytotoxicity (ADCC), or apoptosis [112].

Anti-TNF biology functions by mopping up excess soluble TNF and reducing the endocrine activity of these cytokines. They would bind to TNF complexes to block cell-to-cell contact and/or trigger reverse signalling, lastly acting as agonists on Fc receptor (FcR)-expressing cells as they are fused to human IgG1 [110]. However, most of the previous studies on TNF antagonism focused on the inflammatory cases and particularly on rheumatoid arthritis; consequently there are no experimental studies on breast cancer in this context. As inflammation is known as a significant component in cancer progression and the microenvironment of cancer is controlled by inflammatory cells [113], we estimate that TNF antagonism is able to modify breast cancer cells' signalling cascades inducing cell division, migration, differentiation, or death depending on their expression markers and secreted cytokines.

**Figure 5:**
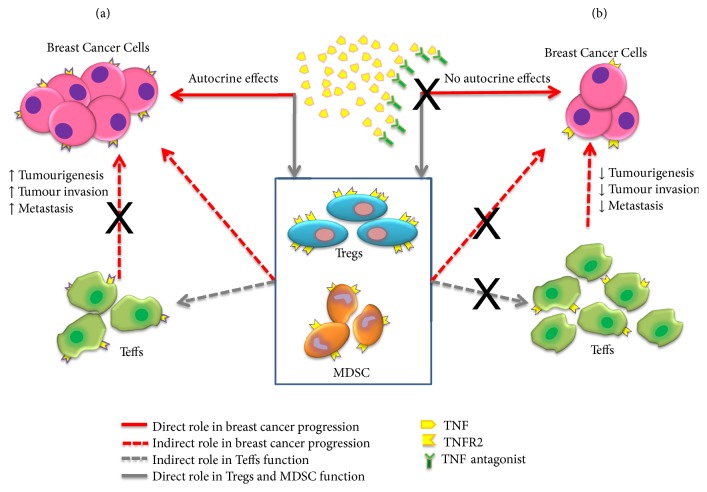
Role of TNF-TNFR2 in the progression of breast cancer and the potential role of TNF antagonists in competing with TNFR2 by mopping up excess soluble TNF and binding on the membrane-bound TNF. (a) TNFR2 is expressed on immune cells and tumour cells in cancer microenvironment. Instead of apoptosis, TNFR2 induces malignant transformation and tumour proliferation by sTNF that activates TNFR2 to enhance Tregs, cancer cells, and MDSC. Therefore, TNFR2 is implicated in enhancing tumour progression either by maintaining cancer microenvironment (immune responses) and enhancing cancer immune evasion, or by inducing cancer cells survival and proliferation [116]. TNFR2 was implicated in promoting the progression of breast cancer via stimulation of AKT signalling pathway which protects against DNA damage and, consequently, enhances proliferation, CAF induction, angiogenesis, and carcinogenesis. Further, a positive association had been reported between TNFR2 expression and its prognosis in terms of size of tumour, higher pathological grade, advanced clinical stage, and dampened doxorubicin resistance [95, 102]. (b) We hypothesized that TNF antagonists would modify breast cancer cells' signalling effects that lead to division, migration, differentiation, or death by assessing their expression markers and secreted cytokines.

Taken together, these findings suggest that targeting TNF-TNFR2 interaction with pharmacological agents, in an attempt to reduce the number and function of Tregs while enhancing the function and number of Teffs, could provide stronger immune responses against cancer cells and serve as a promising cancer therapeutic approach [55, 114]. On the other hand, studies have also shown that TNF-TNFR2 axis enhances the activation of myeloid-derived suppressor cells (MDSCs) and Tregs suppressive cells that promote tumour immune escape [56, 57]. Furthermore, TNFR2 accelerates the programmed death of macrophages for clearing cancer cells [115]. Thus, TNFR2 plays both direct and indirect role in cancer progression (Figure 5) [116]. We can summarize the pathways of these roles as follows: (1) direct effect of TNF in cancer progression modulated by TNF-TNFR2 axis breast cancer cells itself and (2) indirect effect of TNF in cancer progression modulated by TNF-TNFR2 on Tregs and MDSC which ultimately increase tumourigenesis, tumour invasion, and metastasis. TNF-TNFR2 effects are more prominent on Tregs compared to Teffs as these receptors are preferentially expressed by Tregs.

## 5. Nanomedicine in Targeting TNF-TNFR2 Axis

**Figure 6:**
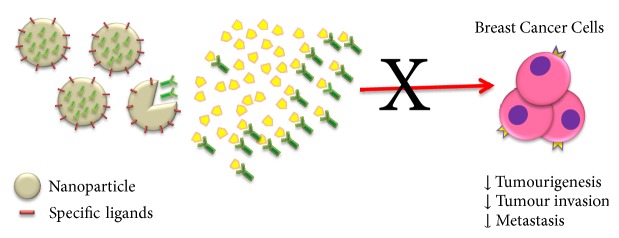
Nanoparticles are expected to serve as an efficient tool to deliver TNF antagonists or even to directly regulate TNF-TNFR2 interactions in breast cancer cells that leads to immunological cascades as observed in Figure 5(b).

As we discussed before, nanomedicine platforms offer a variety of potentially efficient solutions for the development of immunotherapeutic agents that can be exploited for breast cancer treatment [117]. As mentioned before, the first study back in 2008 utilized conjugate gold nanocages with antiepidermal growth factor receptor (anti-HER2) monoclonal antibodies to target breast cancer cells. The targeted cells with the immuno–gold nanocages responded directly to pulsed near-infrared laser irradiation and the mortality rate of cells increased in line with increasing time of exposure till 5 min and became fixed. This study provided significant details regarding the best dosage of immuno–gold nanocages and other information about the parameters of the laser irradiation in breast cancer treatment [66]. Although there are numerous applications of nanoparticles in modulating immune response [39], only a few experimental studies have developed different nanomedicine applications in breast cancer therapeutic research (Table 3). As mentioned earlier, to date there is no study investigating the involvement of nanoparticles in TNF antagonist or in regulating TNF-TNFR2 interactions on breast cancer. Polystyrene nanoparticles, which exhibit various immunological effects in the lung [118], have been used to selectively activate lung TNFR2+ cells, preferentially TNFR2 expressing Tregs. This aforementioned study is the first to show that TNFR2 can be targeted by nanoparticles for therapeutics application in lung diseases [119]. Thus, nanoparticles are expected to serve as an efficient tool to deliver therapeutic agents (including TNF antagonist) or even to directly regulate TNF-TNFR2 interactions in breast cancer cells. Therefore, we hypothesized that the utilization of nanoparticles with specific ligand would alter the function of breast cancer cells (e.g., uptake capacity or downregulation of membrane-bound TNF) and mediate TNF-TNFR2 signal that leads to distinct immunological effects, such as expansion of breast cancer cells, cytokines secretion, or survival capacity (Figure 6).

## 6. Future Directions

Studies have demonstrated that TNF-TNFR2 axis is implicated in the suppression of immune response and affects tumour progression and invasion by its oncogenic roles, which results in enhanced proliferation, angiogenesis, and migration of breast cancer. This receptor is also responsible for enhancing the proliferation and activation of Tregs and MDSCs, thus promoting tumour immune escape. Hence, neutralisation of TNF as well as TNFR2 by using TNF-antagonist drugs might be an effective therapeutic strategy for breast cancer cells. However, no studies to date have investigated the modulation of TNF antagonists targeting TNF-TNFR2 axis and their immunoregulation on breast cancer cells, while the potential of nanoparticles to mediate these effects in breast cancer is still unknown. The field of nanomedicine has provided the possibility of targeting the TNF-TNFR2 axis to not only deliver therapeutic drugs to targeted sites but also restore the immune response to suppress the cancer cells. Based on accumulating evidence suggesting that tumour progression is governed not only by genetic changes intrinsic to cancer cells but also by environmental factors, future studies might use nanoparticles as a model for inert environmental stimuli. In summary, nanoparticles have the potential to be used as drug delivery vehicle in the future for nanomedicine development in breast cancer therapy. Therefore, future studies should investigate how the presence of nanoparticles with specific characterisation would alter the function of breast cancer cells via TNF-antagonist effects by TNF-TNFR2 signal and their contribution to distinct immunological effects.

## Figures and Tables

**Table 1:** The most well studied nanocarrier systems.

Type of nanocarrier	References
Liposomes	[8–12]
Dendrimers	[13–15]
Polymer-based platforms	[16–18]
Superparamagnetism nanoparticulates	[19, 20]
Gold nanoshells	[21–23]
Carbon-60 fullerenes	[24–26]
Nanocrystal	[27–29]
Silicon and silica-based nanoparticle	[30–32]

**Table 2:** The biological subtypes of breast cancer.

Subtypes	Estrogen receptor (ER)	Human epidermal growth factor receptor-2 (HER2)	Ki-67 protein	Progesterone receptor (PR)	Comment
Luminal A Luminal B	Positive Positive	Negative Positive or Negative	Low High	Positive or Negative Positive or Negative	In comparing luminal A and B, luminal A is reported to be growing slower than luminal B, which means best prognosis in luminal A cancer; Ki-67 helps in monitoring how fast tumours grow.

Triple-negative (basal-like)	Negative	Negative	-	Negative	It is defined as basal-like breast cancer and is more common among young women especially with *BRCA1* gene mutations.

HER2-enriched	Negative	Positive	-	Negative	This cancer is growing faster than luminal cancers but with worse prognosis.

Normal-like	Positive	Negative	Low	Positive or Negative	Although ‘normal-like' is similar to luminal A, its prognosis is worse than luminal A.

**Table 3:** List of the studies on nanomedicine development in breast cancer therapeutic research.

Study	Experiment platform	Type of NPs	Conjugated biomolecules	Targeting pathway	Findings
Moses et al., 2016 [35]	*In vitro* on: MCF-7, MDA-MB-231 (breast cancer human cell lines) and MCF-10A (healthy human cells)	AuNPs	Extracts from* Commiphora myrrha* and *Boswellia sacra*	Inducing cytotoxicity	Cytotoxicity in both breast cancer cell lines was more aggressive without harm to healthy cells

Swanner et al., 2015 [58]	*In vitro* on MCF-7, MCF-10A, MDA-MB-231, 184B5, BT-549, and SUM-159 human cells	AgNPs	-	Oxidative stress and DNA damage	AgNPs led to selective cytotoxicity and radiation dose-enhancement effects in breast cancer cells as a self-therapeutic agent

Devulapally et al., 2015 [59]	*In vivo *(animal model) and *in vitro *on MDA-MB-231 human cells	PLGA-b-PEG polymer NPs	Antisense-miR-21 and antisense-miR-10b	Targeting metastasis and antiapoptosis by multitarget antagonisation of endogenous miRNAs	There was a substantial reduction in tumour proliferation at very low dose and 40% reduction in tumour proliferation compared to control

Shu et al., 2015 [60]	*In vivo *(animal model) and *in vitro *on MDA-MB-231 human cells	RNA NPs based on pRNA 3-way-junction (3WJ)	Anti-miR-21	Targeting metastasis and antiapoptosis by multitarget antagonisation of endogenous miRNA	Confirming the potential role of RNA NPs in miRNA delivery in cancer therapeutics

Liu et al., 2014 [61]	*In vivo* and *in vitro* (animal model) on SUM149, BT549, and MCF-10A cells	PEG-PLA NPs	siRNA	Targeting of cyclin-dependent kinase 1 (CDK1) by siRNA induces decrease of cell viability, enhances cell apoptosis	Tumour progression has been suppressed in mice without causing any systemic toxicity, and without activating the innate immune response

Deng et al., 2014 [62]	*In vivo *(animal model) and *in vitro *on MDA-MB-231 human cells	Hyaluronic acid-chitosan NPs	DOX and miR-34a	Suppressing the expression of anti-apoptosis proto-oncogene Bcl-2 and non-pump resistance in tumour cells by DOX. Also, miR-34a plays an intracellular role via targeting Notch-1 signaling which leads to inhibition cancer cell migration	The delivery of miR-34a and DOX has effects on tumour suppression

Deng et al., 2013 [63]	*In vivo *and *in vitro* on MDA-MB-468 animal model cells	Layer-by-layer nanoparticles	siRNA	Targeting of multidrug resistance protein 1 by siRNA enhances DOX efficacy and led to decrease in tumour volume	Increase of DOX efficacy led to decrease of tumour volume with no observed toxicity compared to the control treatments

Wang et al., 2011 [64]	*In vitro* on SK-BR3 and MDA-MB-468 human cells	AuNCs	Herceptin	Targeting and nuclear localization in ERBB2 overexpressing breast cancer cells	AuNCs were able to enter the cell nucleus and promoted the competency of Herceptin drug

Dreaden et al., 2009 [65]	*In vitro* on MDA-MB-231 and MCF-7 human cells	Plasmonic AuNPs	Tamoxifen-PEG-Thiol	Targeting estrogen receptor positive breast cancer cells	A high degree of perinuclear and cytoplasmic localization of the targeted particles, with increased potency and selective intracellular delivery of tamoxifen

Au et al., 2008 [66]	*In vitro* on SK-BR-3	Gold nanocages	Anti-HER2	Targeting of the epidermal growth factor receptor which is overexpressed on breast cancer cells	Optimal parameters of nanocages required to achieve cellular damage and increase percentage of dead cancer cells

Gradishar et al., 2005 [67]	Clinical trial on metastatic breast cancer patients	Albumin NPs	Paclitaxel	Paclitaxel is a chemotherapy drug, and it works based on antineoplastic/cytotoxic mechanism.	Nanoparticle albumin-bound paclitaxel demonstrated greater efficacy and a favourable safety profile compared with standard paclitaxel.

NPs: nanoparticles; AuNPs: gold nanoparticles; AgNPs: silver nanoparticles; DOX: doxorubicin; PLGA: poly(lactic-co-glycolic acid); PEG: poly (ethylene glycol); AuNCs: gold nanoclusters.

## Abbreviations

TNF: Tumour necrosis factor
TNFR: TNF receptor
mTNF: Membrane TNF
sTNF: Soluble TNF
TACE: TNF-converting enzyme
Tregs: Regulatory T cells
Teffs: Effector T cells
NK: Natural killer
IL: Interleukin
HER: Human epidermal growth factor receptor
ER: Estrogen receptor
AuNPs: Gold nanoparticles
AgNPs: Silver nanoparticles
BRCA: Breast cancer
CDK: Cyclin-dependent kinase
DOX: Doxorubicin
HA: Hyaluronic acid
CS: Chitosan
DD: Death domain
NF-*κ*B: Nuclear factor kappa B
RIP: Receptor-interacting protein
TRADD: TNFR-associated DD
FADD: Fas-associated DD
cIAP: Cellular inhibitor of apoptosis protein
TRAF: TNFR-associated factor
PARP: Poly (ADP-ribose) polymerase
MAPK: Mitogen-activated protein kinases
JNK: c-Jun N-terminal kinase
PI3K: Phosphoinositide 3-kinase
CAF: Cancer-associated fibroblast
MDSCs: Myeloid-derived suppressor cells
AP: Activator protein
IBD: Inflammatory bowel disease
SpA: Spondyloarthritis
RA: Rheumatoid arthritis
Fc: Fragment crystallisable
FcR: FC receptor
ADCC: Antibody-dependent cell-mediated cytotoxicity.
